# The State of the Art in the Treatment of Actinic Keratosis and Field Cancerization: A Narrative Review

**DOI:** 10.3390/jpm15090421

**Published:** 2025-09-03

**Authors:** Andrea Paradisi, Enrico Bocchino, Maria Mannino, Giulio Gualdi, Alessandra D’Amore, Daniele Omar Traini, Ketty Peris

**Affiliations:** 1 Dermatologia, Dipartimento di Medicina e Chirurgia Traslazionale, Università Cattolica del Sacro Cuore, 00168 Rome, Italy; enrico.bocchino01@icatt.it (E.B.); alessandra.damore@policlinicogemelli.it (A.D.); danieleomar.traini01@icatt.it (D.O.T.); ketty.peris@unicatt.it (K.P.); 2 UOC di Dermatologia, Dipartimento di Scienze Mediche e Chirurgiche, Fondazione Policlinico Universitario A. Gemelli—IRCCS, 00168 Rome, Italy; 3 Department of Medicine and Aging Science and Dermatologic Clinic, University “G. D’Annunzio”, 66100 Chieti, Italy; giuliogualdi@libero.it

**Keywords:** actinic keratosis, treatment, field cancerization

## Abstract

Actinic keratosis (AK) is considered the early phase of a squamous cell carcinoma (SCC) and represents one of the most common epithelial skin lesions, with an estimated global prevalence of approximately 14%. An estimated annual risk of progression has been reported with a range from 0 to 0.53%. Although spontaneous regression of individual AK lesions has been described in approximately 23% of cases, the frequent presence of multiple lesions, usually in the broader context of field cancerization, significantly diminishes the likelihood of regression and contributes to a higher cumulative risk of progression to SCC. The aim of the present narrative review was to provide an overview of the current evidence of the most effective available lesion-directed and field-directed treatments for actinic keratoses, on the personalized, combined, or sequential approach, as well as on the emerging therapeutic options.

## 1. Introduction

Actinic keratosis (AK) is considered the early phase of a cutaneous squamous cell carcinoma (cSCC) and represents one of the most common epithelial skin lesions, with an estimated global prevalence of approximately 14% [1].

Risk factors for AK development include age >45 years, male gender, a history of non-melanoma skin cancer, outdoor work for more than 6 h a day, skin type I/II, solar lentigines, immunosuppression, use of tanning beds, baldness, use of photosensitizing drugs in chronic therapies, alcohol consumption and immunosuppression [2,3]. In particular, solid organ transplant recipients treated with chronic immunosuppressive therapy have an 80% lifetime risk of developing AK and a 30% risk of having at least five AKs [4].

Clinically, AK presents as an erythematous papule or plaque, scaly, usually <1 mm thick, located in sun-exposed areas; it can be asymptomatic, pruritic, or occasionally tender to palpation [5]. AKs are frequently classified according to the Olsen grading system, wherein grade I lesions are characterized by minimal hyperkeratosis, being more readily appreciable by tactile examination than visual inspection; grade II lesions exhibit moderate hyperkeratosis with both clear visual demarcation and palpable thickening; and grade III lesions demonstrate pronounced hyperkeratosis, often with a verruciform or markedly indurated surface architecture [2,5]. Diagnosis is essentially clinical and dermoscopic and may be aided by noninvasive diagnostic techniques, such as confocal microscopy and line-field optical coherence tomography (LC-OCT), which have also been applied to monitor treatment efficacy of Aks [6,7,8]. Histological confirmation is required only if progression to invasive cSCC is suspected.

An estimated annual risk of progression has been reported with a range from 0 to 0.53% [2,9]. Although spontaneous regression of individual AK lesions has been described in approximately 23% of cases [10], the frequent presence of multiple lesions, usually in the broader context of field cancerization, significantly diminishes the likelihood of regression and contributes to a higher cumulative risk of progression to cSCC [9,11].

These areas represent a subclinical reservoir of atypical keratinocytes with molecular alterations similar to those found in overt lesions, underscoring the need for therapeutic strategies that address not only clinically visible AKs but also the surrounding UV-damaged field to reduce the risk of malignant transformation.

The aim of the present narrative review was to provide an overview of the current evidence on the most effective available treatments for actinic keratoses and emerging therapeutic options.

## 2. Materials and Methods

This narrative review provides state-of-the-art information on AK treatment, incorporating the best available evidence up to 1 April 2025. Primary sources were sought in the PubMed, Scopus, and Cochrane Library databases. Google Scholar was consulted to broaden the search. Search terms included “actinic keratosis” and “field cancerization” along with keywords related to various therapies and treatment techniques (e.g., cryotherapy, cryosurgery, 5-fluorouracil (5-FU), imiquimod, tirbanibulin). The review includes randomized controlled trials (RCTs), scientific society guidelines, interventional studies, and expert consensus statements. Only papers published in English were considered. Case reports and studies that do not meet quality criteria, defined as lacking peer review, including very small or heterogeneous patient cohorts, or failing to provide clear methodological details or clinically relevant outcomes, were excluded. The inclusion of English-language studies only and the lack of systematic cost-effectiveness analyses may limit the applicability of the findings for clinical decision-making.

## 3. Relevant Sections

### 3.1. General Considerations

The management of AKs encompasses two primary therapeutic strategies: lesion-directed and field-directed approaches [2]. Lesion-directed treatments are designed to target individual, clinically visible lesions. However, the majority of AKs develop within areas of chronic sun damage, referred to as field cancerization. These fields represent zones of photodamage and widespread genetic alterations where numerous atypical keratinocytes coexist, along with both clinically apparent and subclinical lesions. While field-directed therapies are often associated with greater local skin reactions and may be less well tolerated by patients, they are increasingly recognized as essential due to the potential for cSCC to develop from subclinical lesions.

Therapeutic modalities can further be categorized based on the setting of administration. ‘In-office’ treatments are performed under the supervision of a healthcare provider, allowing for real-time adjustment of the procedure based on patient’s response and tolerability. Examples include cryotherapy, photodynamic therapy (PDT), and laser therapy. On the other hand, ‘at-home’ treatments are self-administered by patients according to a prescribed regimen, and include topical agents such as 5-FU, imiquimod, diclofenac and tirbanibulin. While offering convenience and cost-effectiveness, at-home therapies require adherence and proper patient education to ensure efficacy and minimize adverse effects.

Importantly, therapeutic choice also varies with clinical grade: most topical agents (Imiquimod and 5-FU) are licensed for non-hyperkeratotic/non-hypertrophic lesions (Olsen grades I–II), and tirbanibulin is EU-approved specifically for Olsen grade I on the face/scalp [2]; by contrast, hyperkeratotic (Olsen grade III) lesions are generally managed with lesion-directed procedures (curettage and/or cryosurgery) and, when appropriate, sequential regimens combining initial debulking with subsequent field-directed therapy [2].

### 3.2. Lesion-Directed Treatments

#### 3.2.1. Cryosurgery

Cryosurgery involves the application of a cryogenic agent to induce targeted tissue destruction through multiple mechanisms [12]. The primary effect is the rapid freezing of intracellular and extracellular fluids, which disrupts cellular membranes and results in mechanical cell rupture. This process triggers the release of intracellular components and pro-inflammatory cytokines, leading to localized immune activation. In addition, cryo-induced vascular stasis, including thrombosis and microthrombosis, causes ischemic necrosis, further contributing to tissue destruction. Being a destructive technique, cryosurgery prevents histopathological analysis of lesions and should be avoided in cases of uncertain diagnosis.

The most widely available and commonly used cryogenic agent is liquid nitrogen (LN), which can be delivered through various methods. The most common involves application with an LN-soaked cotton swab or a pressurized container that releases a continuous LN spray.

Application techniques reported in the literature vary considerably. One protocol suggests continuous applications of LN cryospray lasting 5–10 s, until complete freezing is achieved [13]. The procedure uses an 18-gauge spray tip held 1–2 cm from the lesion at an angle of 90° to the skin. Treatment is repeated after 120 days. The authors report a 63% and 54% reduction in AK lesions on the face and forearms, respectively. However, this was associated with a high incidence of mild complications such as pain, formation of epidermal blisters, local superinfection, and residual hypopigmentation (58%).

Comparison of the effects of one application cycle versus two consecutive cycles to the same lesions found a high lesion clearance rate after a single cycle (≃80%), without significant differences in the presence of cytological atypia, hyperkeratosis, dyskeratosis, dermal fibrosis, and inflammatory cells in the dermis as assessed by confocal microscopy [14].

Treatment with an LN-soaked cotton swab is both cost-effective and widely available. According to some authors, the commonly used protocol involves direct contact of the swab with the lesion for 10–20 s, until a peripheral frozen rim of 1–2 mm is achieved [15]. At 90 days, a complete response rate of ≃71% and a good-to-excellent cosmetic outcome of ≃81% of cases have been described.

Although LN cryosurgery is a lesion-oriented technique, some authors suggest a broader application of LN to cover the entire field cancerization (or cryopeeling). However, as of today, standardized protocols and robust data on its efficacy and safety are still lacking [16,17].

#### 3.2.2. Ablative Lasers

The use of ablative lasers, most commonly CO_2_ lasers and erbium:YAG lasers, is a highly operator-dependent procedure that is also affected by the lack of standardized protocols for AK treatment. In a small case series, facial resurfacing with a CO_2_ laser resulted in a 92% reduction in AKs at 3 months, without adverse events [18]. However, in a larger sample size the results were less favorable: at 3 months, CO_2_ laser ablation achieved complete clearance of ≃65% of lesions, but recurrence rates at 12 months were ≃78%, substantially inferior to cryosurgery albeit with similar tolerability [15].

In one RCT comparing 5-FU with laser resurfacing, a protocol involving epidermal laser ablation of the entire scalp achieved a clearance rate of ≃94% at 6 months and ≃90% at 12 months [19]. Though superior to the 5-FU outcome, this result was however associated with frequent and significant adverse effects, including erythema, edema, crusting and infection, despite prophylactic antibiotic therapy.

Ablative laser devices may also be combined to Methyl aminolevulinate (MAL) or Aminolevulinic acid (ALA)-PDT as a lesion or field-targeted pre-treatment by (laser-assisted PDT) [20]. An ablative fractional laser has direct cytotoxic effect and creates microscopic vertical channels that may facilitate the penetration and enrichment of ALA or MAL in dysplastic cells, a concept that has been termed “laser-assisted drug delivery”.

In a randomized split-scalp study, 56 patients were treated with ablative fractional CO_2_ laser using a protocol involving 19 W power, 1000 µm spacing, triple stacking, and a scan time of 1800 µs, followed by PDT with MAL [21]. At 6 months, the combination with laser pretreatment showed better outcomes in terms of complete clearance, partial clearance, and lesion-specific clearance rates than PDT alone, as well as a comparable tolerability. These findings are supported by other clinical studies [22,23,24].

### 3.3. Surgery

AKs do not generally require excisional biopsy; however, current guidelines recommend histopathological examination when the diagnosis is uncertain or in cases of treatment resistance, particularly in high-risk areas with an increased likelihood of progression to cSCC, such as the lips, ears, dorsal hands, and lower limbs [2]. In such cases, scalpel excision is recommended to obtain histopathological confirmation and to accurately define the surgical margins.

Curettage is primarily used in combination with other treatments, particularly PDT. The authors question the necessity of curettage as a pre-treatment for PDT, suggesting that PDT may be effective even without it [25,26,27].

### 3.4. Field-Directed Treatments

#### Photodynamic Therapy

PDT is based on photochemical reactions, where activation of a photosensitizing molecule by light leads to the generation of reactive oxygen species (ROS) through energy transfer to oxygen [28]. The ROS induce cellular damage, ultimately resulting in the destruction of targeted cells.

Currently, the most commonly used photosensitizing molecules are 5-aminolevulinic acid (5-ALA) and MAL [29,30]. These compounds, derivatives of ALA, are metabolized into protoporphyrin IX (PpIX), an intermediate in hemoglobin biosynthesis that also has photosensitizing properties. Exogenous application of 5-ALA and MAL induces intracellular accumulation of PpIX, which, upon exposure to a light source, exerts its photosensitizing effect. Neoplastic cells are particularly susceptible to this mechanism due to their metabolic switch, which leads to increased and uncontrolled hemoglobin production as well as enhanced enzyme activity, which sustains the process. MAL, the agent used most frequently in Europe, is available in a cream formulation, typically applied under occlusion to the target area, and is converted to PpIX. Additionally, the disruption of the epithelial barrier characteristic of AKs facilitates vehicle penetration. The other essential component of the photodynamic reaction is light irradiation. This can be achieved through exposure of the treatment area either to natural sunlight, i.e., daylight PDT (dlPDT), or to a predefined-wavelength light source in a clinical setting, i.e., conventional PDT (cPDT) [29,30]. PpIX exhibits multiple absorption peaks in the visible spectrum, leading to photoexcitation and ROS generation. An effective activation range is between 400 and 430 nm, in the blue-violet spectrum, which can be achieved using a blue light source [31]. However, use of this protocol is limited by its shallow penetration depth. In clinical practice cPDT is more performed commonly, using a lamp emitting red light at a wavelength of 630–635 nm, which provides greater tissue penetration.

In practical terms, MAL-cPDT requires cleansing of the treatment area followed by the application of MAL cream under occlusion, which must remain in place for approximately 3 h. Before red light exposure, the cream is gently removed from the treated area and irradiation at a power of 37 J/cm^2^ is performed for about 8 min [32].

The duration of irradiation has recently been questioned by a split-face trial comparing irradiation times of 8 and 4 min. Besides slightly greater pain with the longer irradiation during exposure, the study found comparable efficacy (70% vs. 65%, respectively) and similar adverse event rates at 6 months [33].

Alternative protocols of total light dose and fluence rate were evaluated in a prospective randomized trial, in which Olsen grade I and II AKs were treated with cPDT using BF-200 ALA [34]. The study demonstrated that protocols employing either a halved total dose and/or a reduced fluence rate achieved comparable overall clearance rates at 3 months post-treatment, while significantly reducing both mean and maximum pain intensity in the reduced fluence rate groups, and lowering phototoxicity scores across all modified protocols [34].

Reports of the efficacy of MAL-cPDT vary (AK clearance: 37.1–94.6%) [35,36,37]. Although cPDT is widely available, the requirement for patients to remain in the clinical setting for at least 3 h and the pain experienced during the procedure, particularly when treating AKs, have been reported to limit its applicability and to lead to a preference for dlPDT [31].

Treatment with dlPDT can be initiated soon after MAL application [38]. After cleansing the treatment area and applying a chemical sunscreen, MAL is applied without occlusion. Light exposure should begin within 30 min of photosensitizer application [38]. The shorter the interval between MAL application and light exposure, the lower the perceived pain. Sunlight exposure duration must be at least 2 h: a shorter duration may be ineffective, while a longer duration may increase the incidence of adverse events. A key limitation of dlPDT is the need for optimal weather conditions, as it requires clear days ensuring uninterrupted sunlight exposure while should not be performed during rain [38].

RCTs comparing the efficacy and tolerability of dlPDT and cPDT are few. According to a recent RCT, efficacy was substantially similar (90.0% and 94.6% AK reduction, respectively, 6 months post-treatment) although cPDT was associated with significantly higher pain scores [37]. Similarly, another RCT has reported a comparable efficacy at 3 months (89.2% vs. 92.8%, respectively) and overall higher patient satisfaction, with lower pain for dlPDT [39]. An Italian RCT found a lack of significant difference in efficacy for grade I AKs at 3 months, whereas dlPDT was less effective than cPDT for grade II and III Aks [40]. Nevertheless, the vast majority of patients (88%) still preferred dlPDT. Given the efficacy and tolerability of dlPDT, various indoor systems have been developed to replicate and standardize daylight illumination conditions in an indoor setting. These approaches are collectively referred to as simulated dlPDT (sdlPDT) and include the use of operating rooms or specific devices [41,42]. SdlPDT has shown high efficacy, with direct lesion clearance rates of approximately 85% at 3 months after treatment [42].

A formulation known as BF-200 ALA gel, incorporating a nanoemulsion of 7.8% ALA that enhances tissue penetration, was recently evaluated in a multicenter trial as a field-directed therapy for mild-to-moderate AKs [43]. Following treatment, approximately 91% of patients achieved complete clearance, with 75% and 63% of these maintaining a complete response at 6 and 12 months of follow-up, respectively [43].

### 3.5. Tirbanibulin Ointment

Tirbanibulin 1% ointment is a topical treatment for grade I AKs on the face and scalp, with a recommended application area of up to 25 cm^2^ [44]. Tirbanibulin acts as an antiproliferative agent by binding to intracellular tubulin and inhibiting its polymerization, thereby disrupting cytoskeletal formation during mitosis [45]. The treatment regimen consists of once-daily application for five consecutive days. Adverse events are predominantly mild, with erythema reported in 91% and scaling in 82% of patients [46].

A notable limitation of tirbanibulin is the fact that is approved for areas up to 25 cm^2^. However, a recent multicenter, single-arm study evaluated the safety of extending the application area up to 100 cm^2^ [47]. As with earlier trials, hyperkeratotic and hypertrophic AKs, as well as lesions on the lips, eyelids, and ears, were excluded. Most adverse events were mild to moderate, with severe erythema and severe scaling reported in 5.8% and 8.7% of cases, respectively. Adverse reactions peaked between days 5 and 8 and resolved within two months.

In a single-center trial, tirbanibulin was shown to improve multiple parameters of actinic damage, including facial dyschromia and erythema, suggesting its potential future application for esthetic purposes and overall skin quality enhancement [48].

### 3.6. 5-Fluorouracil

5-FU is a cytostatic antimetabolite structurally similar to thymine. It interferes with thymine utilization, disrupting DNA and RNA synthesis and thereby inhibiting cellular replication [49]. The drug is contraindicated during pregnancy and breastfeeding [50]. Concurrent use with certain antiviral agents such as brivudine and sorivudine should be avoided due to the risk of elevated plasma 5-FU levels from enzymatic competition [51].

Available formulations include 5% cream (with or without 0.005% calcipotriol), 4% cream, and 0.5% cream (with or without 10% salicylic acid). The 4% aqueous cream in peanut oil is approved for grade I and II AKs on the face, ears, and scalp in adults, without surface area limitations. Due to peanut oil content, it is contraindicated in patients with peanut or soy allergies [46].

An RCT comparing 4% and 5% formulations found that daily application for 4 weeks resulted in 80% complete lesion clearance and 100% partial clearance with both concentrations [52]. Skin irritation occurred in 30% of patients, with the 4% formulation demonstrating better tolerability. Real-world studies confirmed similar efficacy, reporting complete clearance in 74.5% of AKs and no recurrences at 6 months in 65% of cases [53]. The most common side effects included moderate erythema (51%) and a burning sensation (moderate in 22%, severe in 1%), both resolving by follow-up [53].

Although adverse events may lead some patients to discontinue treatment with 4% 5-FU prematurely, a dose-ranging study reported high clearance rates of AKs even in patients treated with once-daily applications for two weeks, with improved tolerability [54].

Another RCT comparing 5% 5-FU applied twice daily for 4 weeks to imiquimod (IMIQ), MAL-PDT, and ingenol mebutate showed a therapeutic success rate of 74.7% at 12 months [55]. However, 91% of patients experienced adverse effects, including moderate-to-severe erythema (81.5%), vesicles/blisters (24%), severe pain (16%), burning (21%), and pruritus (61%) [55]. Two weeks post-treatment, moderate-to-severe erythema persisted in 58%, blisters in 21%, and severe pain in 7% of patients.

A 5% 5-FU and 10% salicylic acid (5-FU/SA) formulation is marketed for lesion-directed therapy of grade I and II AKs on the face, scalp, neck, and extremities in immunocompetent adults. Treatment is limited to a maximum of 10 lesions and 25 cm^2^. Applied once daily for up to 12 weeks, it demonstrated 49.5% complete and 69.5% partial clearance at 8 weeks in a phase III trial [56]. Adverse events, though slightly more common than placebo (99% vs. 83%), were mostly mild, including erythema (88.9%), mild pain (69.4%), and irritation (59.3%). Systemic effects such as nasopharyngitis (11.1%) and headache (3.7%) were also reported [56].

Calcipotriol, a vitamin D analog, has recently been tested in combination with 0.5% 5-FU [57]. This formulation may enhance immune-mediated clearance of AKs by promoting thymic stromal lymphopoietin expression and CD4+ T-cell activation [57].

Another combination protocol was evaluated in a single-center, split-face study including AKs of all grades. The regimen consisted of 4% 5-FU applied twice daily for 7 days, followed by a session of dlPDT [58]. This approach demonstrated a slightly higher efficacy compared to dlPDT alone at 12 months, with complete response rates of 79.2% versus 70.4% [58].

### 3.7. Imiquimod

Imiquimod is an immunomodulatory drug that binds to and activates toll-like receptors (TLRs), particularly TLR7, on antigen-presenting cells. This interaction triggers the production of nuclear factor kappa-B and promotes the secretion of pro-inflammatory cytokines, such as tumor necrosis factor-α and interferon-α. Overall, this mechanism enhances cell-mediated antineoplastic activity at the site of application [59].

The most commonly used cream formulations are currently the 5% and 3.75% IMIQ [2]. The 5% cream has been approved to treat AKs on the face and scalp on a maximum surface area of 25 cm^2^ [2].

A randomized study comparing 5% 5-FU, cryotherapy, and 5% IMIQ (applied 3 times weekly for 4 weeks) found a complete clearance rate of 73% for imiquimod-treated lesions, which was sustained at 12 months. This outcome was superior to both comparator arms and was associated with better esthetic results [60]. In an RCT comparing 5% imiquimod, 5% 5-FU, MAL-PDT, and ingenol mebutate, the therapeutic success rate of the IMIQ formulation was 71%, higher than that of MAL-PDT and ingenol mebutate but lower than the one of 5-FU [55]. Adverse events occurred in 85% of cases, mainly consisting of moderate-to-severe erythema (72%), moderate-to-severe crusting (68%), and moderate-to-severe pruritus (62%), which mostly resolved within 2 weeks.

The 3.75% IMIQ cream is approved for the treatment of AKs of the face and scalp over a surface area of up to 200 cm^2^, with a regimen of daily application for 2 weeks followed by a 2-week treatment-free interval and by another 2-week treatment period [2]. Its complete lesion clearance rate was 34.0–35.6% at 8 weeks [61,62]. Adverse events were mainly related to local reactions, including pruritus (9.3%) and pain (9.3%). Systemic adverse events were also reported, such as flu-like syndrome (8.0%), headache (4.9%), and fatigue (4.9%) [61].

### 3.8. Other Topical Therapies

Diclofenac sodium 3% in 2.5% hyaluronic acid gel is approved for the treatment of AKs and field cancerization on face and scalp areas up to 200 cm^2^ and is applied twice daily for 60 to 90 days [2]. Its mechanism of action involves the inhibition of cyclo-oxygenase-2 and the induction of cell apoptosis, likely mediated by caspase activation and the fragmentation of intracellular nucleic acids [63].

Its efficacy and safety have been investigated in several clinical trials. In one comparative trial with ingenol mebutate, the endpoint of complete clearance at the end of treatment was achieved in 23.5% of patients. Adverse events were mild and local, including erythema (11.5%), scaling (4.7%), and pain (3.4%) [64].

In one RCT, 3% diclofenac sodium plus hyaluranon gel was compared with 5% imiquimod cream using the Total Thickness Score to assess the clinical severity of AKs based on palpation and visual inspection. Significant differences between the treatments emerged only at 24-week follow-up, with complete clearance rates of 14.3% and 45%, respectively [65]. Despite the advantage of being applicable to large skin areas, treatment with 3% diclofenac gel appears to have a relatively limited efficacy and is generally not recommended as first-line field therapy; it may be considered when an excellent tolerability profile is prioritized over clearance rates.

Also, topical retinoids (e.g., tretinoin, adapalene, tazarotene) have been studied for AK, given their role in modulating keratinocyte proliferation and differentiation [66]. However, evidence for topical is heterogeneous and mostly older; small, controlled trials reported modest AK count reductions but with frequent irritation, and contemporary guidelines do not recommend topical retinoids as standard AK treatment, because of inconsistent data on efficacy compared with approved field therapies [2,66].

### 3.9. Emerging Therapies

Given the need for a highly individualized approach in managing AKs and the considerable variability in indications among existing treatments, novel active compounds are being investigated to broaden the therapeutic options.

An ongoing multicenter, randomized, open-label phase 2 study is evaluating the safety and efficacy of Bimiralisib 2% gel, a pan-PI3K/mTOR inhibitor, for the treatment of AKs on the face, scalp, and/or back of the hands [67]. The product is applied to target lesions for either 2 or 4 weeks. Results are expected later this year.

Resiquimod elicits its effect by binding to and activating TLR7/8, which are primarily expressed on immune cells such as dendritic cells, macrophages, and B lymphocytes [68,69]. Their activation triggers the nuclear translocation of the transcription factor NF-κB and the induction of other transcriptional pathways, leading to an increased production of cytokines, particularly interferon-α, and promoting Th1-type immune responses. Resiquimod gel has been evaluated in a prospective, randomized, partly placebo-controlled, double-blind phase II dose-finding study to assess its safety, tolerability, and efficacy in patients with multiple Aks [69]. However, head-to-head comparisons with other topical therapies have not yet been published.

Colchicine (COL) is a natural alkaloid extracted from the corm of Colchicum autumnale. Its mechanism of action is based on the inhibition of mitotic spindle microtubule polymerization during cell division. In a randomized, open-label, self-controlled clinical trial, 0.5% COL cream or 5% 5-FU cream was applied on forearm skin twice daily for 7 days [70]. After 14 days, complete and partial remission were recorded in 17% and 78% of patients treated with COL, respectively, although adverse events (such as erythema, desquamation, and crusting) were more intense than those reported in the 5-FU group.

## 4. Conclusions

**Figure 1:**
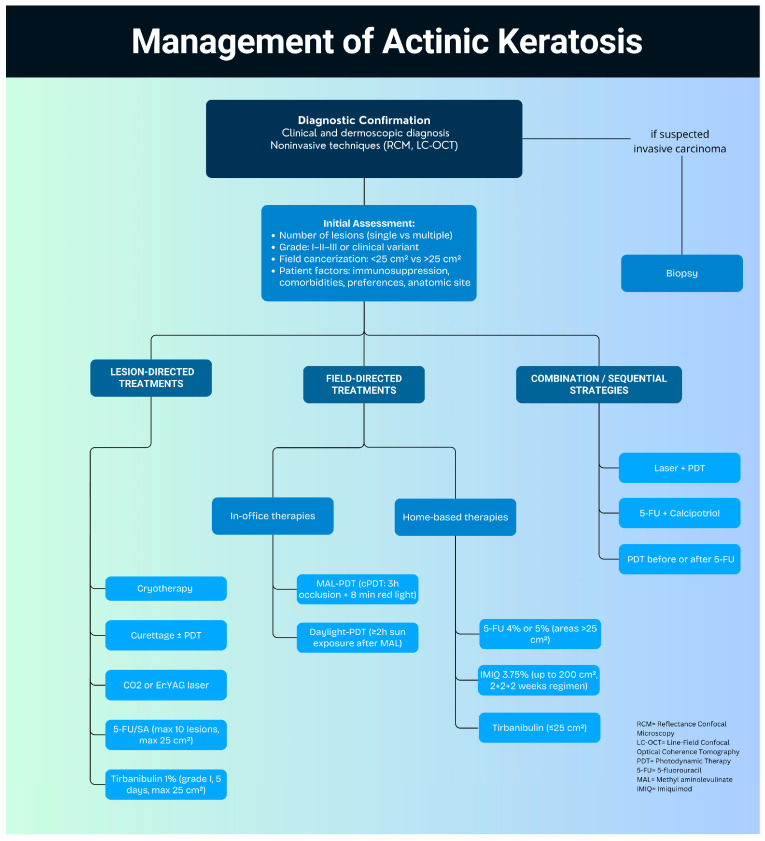
Proposed algorithm for the management of actinic keratosis.

In conclusion, a wide range of effective therapies are available for managing AKs, each offering distinct advantages and limitations in terms of efficacy, tolerability, and practicality. A practical proposal for a therapeutic algorithm for AKs is illustrated in Figure 1. Achieving optimal outcomes requires a personalized, patient-centered approach that considers lesion grade, extent of field cancerization, patient preferences, local adverse reactions, treatment cost and duration, comorbidities, lifestyle factors, and individual tolerability. Tailoring therapy to the unique needs of each patient not only improves adherence but also enhances clinical outcomes and supports long-term disease control and prevention [71,72].

Emerging evidence highlights the value of multimodal strategies, such as combination or sequential therapies, that integrate different treatment modalities for synergistic benefit [73]. Examples include laser-assisted PDT, the novel 5-FU/calcipotriol combination, or a practical sequential approach combining a physical treatment for single hyperkeratotic lesions (i.e., cryotherapy or ablative CO_2_ laser) followed one month later by a field cancer treatment. A very recent randomized intraindividual comparative clinical trial [74] compared the efficacy and safety of sequential treatment with cryotherapy followed by 1% topical tirbanibulin versus cryotherapy monotherapy, showing an improved lesion clearance and a reduction in new AK development for the sequential cryotherapy and tirbanibulin approach.

When adapted to individual patient profiles, the sequential combined approaches can boost efficacy, broaden treatment scope, and help overcome the limitations of monotherapy [75].

Nevertheless, to date, relatively few studies have investigated or established standardized protocols for personalized, combination, and rotational treatment strategies of AK, particularly for patients with extensive field cancerization [75]. Ultimately, the future of AK and field cancerization management lies in dynamic, adaptive strategies that integrate diverse therapeutic options, precisely tailored to each patient’s unique clinical presentation and personal needs, ensuring sustained efficacy and enhanced quality of life.

## Data Availability

No new data were created or analyzed in this study. Data sharing is not applicable to this article.
